# Refractory Hyperactive Delirium Without Intravenous Access: A Case Report

**DOI:** 10.7759/cureus.45694

**Published:** 2023-09-21

**Authors:** George Sun, Vasundhara Acharya, Kevin J Min

**Affiliations:** 1 Department of Anesthesiology, Thomas Jefferson University Hospital, Philadelphia, USA

**Keywords:** delirium in icu, difficult intravenous access, hyperactive delirium, delirium, refractory delirium

## Abstract

Refractory delirium is a complex, often underdiagnosed, and difficult-to-treat phenomenon. It poses significant challenges to healthcare providers, especially in patients without prior intravenous access. In extreme cases, anesthetic management may be needed to treat refractory delirium. Here, we present a unique case of postoperative hyperactive refractory delirium in a patient without intravenous access, ultimately requiring anesthetic management for resolution.

## Introduction

The Diagnostic and Statistical Manual of Mental Disorders, 5th Edition (DSM-V), defines delirium as a state of “acute and fluctuating disturbances of consciousness with reduced ability to focus, maintain, or shift attention, accompanied by changes in cognition and perceptual disturbances” [1]. Most commonly, delirium occurs in elderly patients within a critical care setting, with studies showing up to 80% of patients in the intensive care unit experiencing some form of delirium [2]. While the etiology of delirium is often multifactorial, early recognition of risk factors, avoidance of triggers, and a conservative approach (ambulation, proper sleep hygiene, avoiding unnecessary procedures, and spending time with family) are usually adequate for management [3-4]. In some cases, however, a patient’s delirium symptoms may persist despite conservative therapy, commonly known as refractory delirium. Commonly, refractory delirium is defined in literature as the persistence of delirium symptoms despite adequate treatment without impairing consciousness. While no definitive treatment for refractory delirium has been identified, it is typically managed with antipsychotics or dexmedetomidine for sundowning [5-6]. Electroconvulsive therapy may also be considered as a treatment for protracted refractory delirium [7]. Here, we present a unique case of severe postoperative delirium refractory to medical management that ultimately required anesthesia consultation for management. Written patient consent and Health Insurance Portability and Accountability Act authorization were obtained for this case report.

## Case presentation

**Figure 1:**
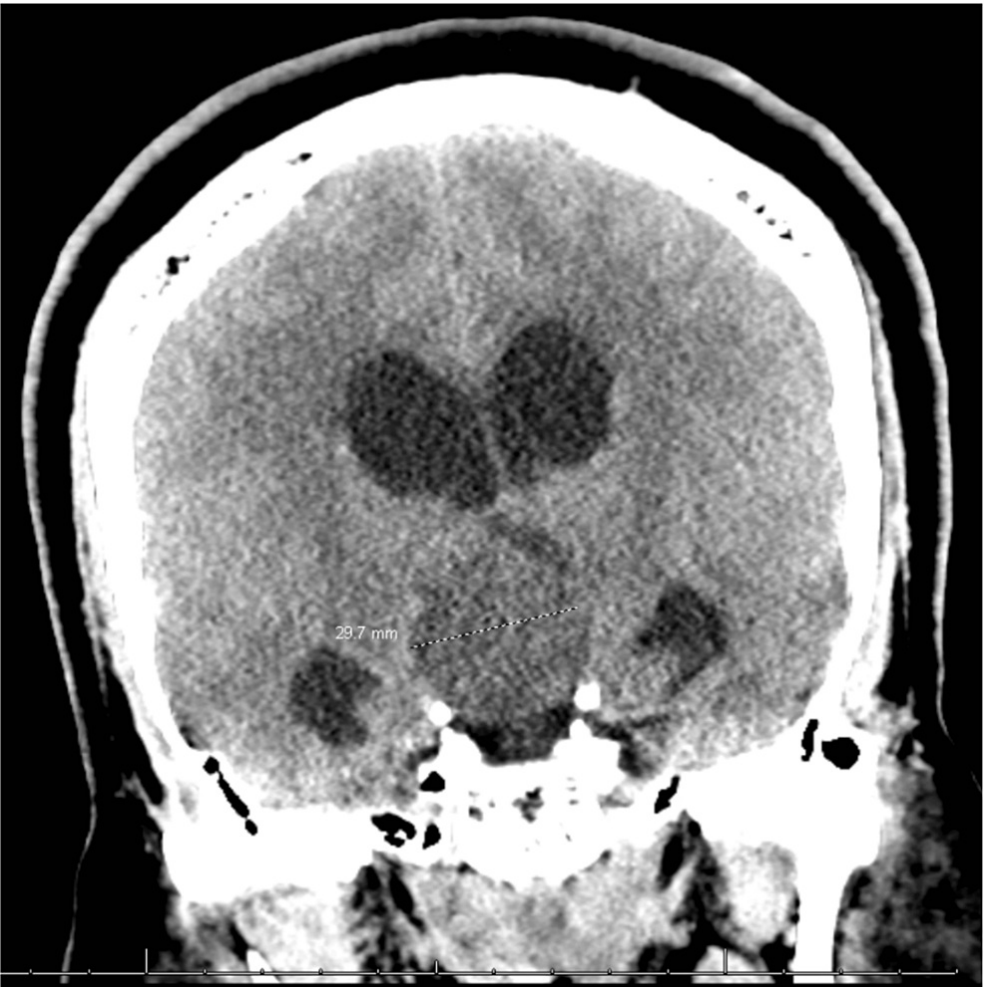
Heterogenous suprasellar mass containing mixed attenuation components with mild ventriculomegaly seen on computer tomography imaging of the head without contrast.

A 28-year-old male, with a past medical history of diabetes and no prior psychiatric history, presented as a transfer from an outside hospital for altered mental status, lethargy, and hyperglycemia. He was found to have obstructive hydrocephalus from a large, suprasellar craniopharyngioma (4.3 cm x 4.6 cm x 3.6 cm) (Figure 1). Otherwise, history was negative for alcohol abuse and illicit drug use.

One week after admission, the patient underwent uncomplicated transsphenoidal debulking of the craniopharyngioma with neurosurgery. Three days postoperatively in the intensive care unit (ICU), the patient, who had earlier been calm and cooperative, had severe delirium with combativeness toward staff and was uncooperative with medical care. At that time, the patient was aggressive toward staff, had climbed out of bed, and began attempting to enter other patient rooms. As the patient was unable to be redirected, security was subsequently called. He ultimately required four-point restraints and sedation with increasing doses of dexmedetomidine (up to 1.5 mcg/kg/hr), three doses of lorazepam 2 mg, two doses of midazolam 4 mg, and two doses of intravenous (IV) haloperidol 5 mg with minimal effect. Despite four-point restraints, the patient pulled out all his IV access including a central line and arterial line. Anesthesia was called stat for consultation given his lack of vascular access and increasing combativeness. Upon arrival, the patient was physically and verbally combative, flailing his arms and legs, and was being held down by four security guards.

After extensive discussion with the ICU team, it was concluded that the patient would need to undergo optimization for IV and arterial access and would likely require intubation to prevent further harm to himself and others, as well as to achieve an adequate level of sedation for line placement. The team was also informed that the patient was known to have poor veins and had removed his right upper extremity PICC line one hour prior to arrival, so the decision was made to proceed with intraosseous access. An intraosseous kit, a video laryngoscope, an Ambu bag, an intubation kit, and ketamine (500 mg) were prepared. Attempts were made to preoxygenate the patient, but the patient was too agitated to tolerate the mask. Thus, ketamine (5 mg/kg) was administered intramuscularly. After two minutes, the patient displayed visible signs of decreased combativeness and increased sedation before the anesthesia team proceeded with an uncomplicated right lower extremity intraosseous line and preoxygenation with a 30-degree head elevation. Once the intraosseous (IO) line was secured and the patient was adequately preoxygenated, induction was performed using propofol (200 mg) and rocuronium (100 mg) to minimize hypertension and rapidly optimize the patient for intubation. Mask ventilation was not conducted, and the patient was directly intubated via glidescope.

After intubation, a triple lumen central venous catheter was placed for more permanent intravenous access, as well as an arterial line for blood pressure monitoring after intracranial surgery. Sedation was maintained with a propofol infusion for the night. The patient was extubated the following morning. Further investigation into the underlying cause of his delirium, including sepsis and metabolic derangements (renal function, liver function, thyroid function, electrolytes, prolactin, procalcitonin, blood and urine studies) were negative. Postoperative head CT and MRI brain showed stable postsurgical changes with no evidence of residual tumor or enhancement in the surgical bed. Given his continued agitation, lorazepam and haloperidol were added, but with minimal effect.

Despite treatment and 1:1 observation, the patient still displayed symptoms of delirium 11 days postoperatively. Over the course of the next two weeks, his cognitive ability improved with near-complete resolution of his psychomotor agitation.

## Discussion

Here, we present an interesting case report of a 28-year-old with a case of severe postoperative delirium refractory to medical treatment, ultimately requiring general anesthesia for resolution. In determining treatment, it is essential to delineate between delirium and other causes of acute confusion, including dementia, metabolic derangements, and medication toxicity. Delirium itself may present broadly as hyperactive, hypoactive, or mixed variety. In evaluating our patient using the confusion assessment method for the intensive care unit (CAM-ICU) guidelines (Table 1), the patient was found to meet all four inclusion criteria, confirming our diagnosis of delirium, predominantly hyperactive type. In our case, the patient’s delirium was likely multifactorial, including due to his brain craniopharyngioma and age.

**Table 1:** Confusion assessment method for the intensive care unit (CAM-ICU).

Confusion Assessment Method for the Intensive Care Unit (CAM-ICU)
The Presence of Delirium Requires Features 1 and 2 and Either 3 or 4
1. Acute Onset or Fluctuation of Mental Status
2. Inattention
3. Altered Level of Consciousness
4. Disorganized Thinking

Refractory delirium is a serious and often underdiagnosed pathology. While the management of refractory delirium is often multimodal, current clinical guidelines do not exist around the best treatment protocols. Isolated case reports, however, have shown limited evidence for several agents. In one such case, olanzapine was used as adjuvant therapy in refractory delirium; in another study, there was evidence for the use of the atypical antipsychotic agent aripiprazole showing better treatment outcomes with fewer side effects [5,8]. Other agents, such as valproic acid, physostigmine, and quetiapine, have been shown to have limited success in treating ICU delirium [9-10].

In our case, the concomitant use of multiple pharmacologic agents, including quetiapine, olanzapine, risperidone, valproic acid, and ultimately dexmedetomidine, was required to resolve the patient’s refractory delirium. In addition, the complete impact of the patient’s craniopharyngioma is unknown given the extensive involvement of the bilateral hypothalami. Anesthesia is typically reserved for situations where all other conservative measures have failed, and the patient’s ongoing agitation and distress pose a significant risk to their safety or the safety of others [11].

Anesthetic management of refractory delirium remains delicate since anesthesiologists must balance the need for deeper levels of sedation with the patient’s ability to maintain a patent airway and protect themselves from aspiration. Given that less sedating agents had been tried without success, it was clear that a more potent sedative was required to achieve adequate conditions for airway management and IV and arterial access.

Since the patient had no IV access, oral and intramuscular (IM) routes were the only viable methods of drug delivery. IO access without sedation was considered but deemed suboptimal given that the patient was already difficult to control with the assistance of multiple hospital personnel. Multiple intramuscular sedative agents were considered, including, haloperidol, midazolam, dexmedetomidine, fentanyl, succinylcholine, and ketamine. Given the QT-prolonging side effects of haloperidol and the risk for extrapyramidal side effects with the likely needed high dosage, haloperidol was ultimately not chosen. Midazolam, olanzapine, and haloperidol also have much slower onsets according to a randomized control trial by Chan et al. [12]. Midazolam has also been shown to significantly decrease upper airway tone, increasing the risk for airway intervention and aspiration [13]. Ultimately, IM ketamine was chosen due to its ability to induce a deep level of sedation while maintaining upper airway muscle tone and respiratory drive. This was especially important since the patient had recently eaten less than two hours ago and, thus, was at risk for aspiration. Using ketamine, a level of sedation was achieved to where the patient was able to tolerate the IO access placement and the mask for preoxygenation, without the need for jaw thrust or airway adjuncts such as oral airway and nasal trumpets.

Rapid sequence intubation without mask ventilation was performed after adequate preoxygenation to minimize the patient’s risk of aspiration. Cricoid pressure maneuver was not performed on this patient before or during intubation in concordance with findings by Birenbaum et al., which showed no reduction in the risk of aspiration and an increase in the time to intubation and the rates of encountering difficult intubations [14]. In case of patient desaturation before achieving a secure airway, we were prepared to mask and ventilate with cricoid pressure to minimize gastric insufflation and risk of aspiration. Ultimately, however, this was not necessary as patient intubation proceeded without issue.

An ideal situation supports rapid control of agitation with subsequent maintenance of sedation, especially in agitated patients without IV access. Medication choice and route of administration are important considerations. Prior studies have shown ketamine as a viable agent in combative patients (low dose, minimal side effects, and ability to maintain respirations despite sedation and upper airway muscle tone), yet ketamine administration does not decrease delirium despite previous thoughts to the contrary [15]. Especially in agitated patients without IV access, ketamine may be safely administered for rapid sedation with excellent efficacy and few clinical side effects, making it an ideal sedative agent [16]. In comparison to other intramuscular agents, ketamine provides a significantly faster time to adequate sedation [17]. If general anesthesia is needed for management, it may be beneficial to use cricoid pressure during mask ventilation, as it has been shown to decrease gastric insufflation, which decreases aspiration risk, especially in patients who have not been nil per os (NPO) [18]. In emergency scenarios where IV access is difficult, the next course of action is to proceed with an IO line [19]. These lines are quick and reliable with few risks.

## Conclusions

This report emphasizes the emergency treatment of refractory postoperative delirium with general anesthesia and the importance of continued awareness among providers regarding early recognition and treatment of postoperative refractory delirium. This condition poses significant challenges and requires specialized care to address, especially in patients without IV access. Timely recognition and intervention are paramount for patient well-being. Through a comprehensive multimodal approach, with initial sedation with intramuscular agents and subsequent maintenance intravenously or intraosseously once access has been established, the impact of refractory delirium can be minimized. However, it is essential to emphasize the importance of careful patient assessment, proper dosing, and vigilant monitoring when using intramuscular agents as agitated patient safety is paramount. Healthcare providers should be well-trained in the administration of these agents and remain observant in monitoring for potential adverse effects. As medical knowledge and best practices evolve, intramuscular agents' role in managing agitated patients may expand, providing a valuable tool for healthcare providers in delivering timely and safe care in challenging situations.
